# An Indoor Mobile Location Estimator in Mixed Line of Sight/Non-Line of Sight Environments Using Replacement Modified Hidden Markov Models and an Interacting Multiple Model 

**DOI:** 10.3390/s150614298

**Published:** 2015-06-17

**Authors:** Jingyu Ru, Chengdong Wu, Zixi Jia, Yufang Yang, Yunzhou Zhang, Nan Hu

**Affiliations:** School of Information, Northeastern University, Shenyang 110819, China; E-Mails: jiazixi@ise.neu.edu.cn (Z.J.); Yang_Yufang@126.com (Y.Y.); zhangyunzhou@ise.neu.edu.cn (Y.Z.); hunan0806@163.com (N.H.)

**Keywords:** wireless sensor networks, localization, non-line-of-sight, hidden Markov models, interacting multiple model

## Abstract

Localization as a technique to solve the complex and challenging problems besetting line-of-sight (LOS) and non-line-of-sight (NLOS) transmissions has recently attracted considerable attention in the wireless sensor network field. This paper proposes a strategy for eliminating NLOS localization errors during calculation of the location of mobile terminals (MTs) in unfamiliar indoor environments. In order to improve the hidden Markov model (HMM), we propose two modified algorithms, namely, modified HMM (M-HMM) and replacement modified HMM (RM-HMM). Further, a hybrid localization algorithm that combines HMM with an interacting multiple model (IMM) is proposed to represent the velocity of mobile nodes. This velocity model is divided into a high-speed and a low-speed model, which means the nodes move at different speeds following the same mobility pattern. Each moving node continually switches its state based on its probability. Consequently, to improve precision, each moving node uses the IMM model to integrate the results from the HMM and its modified forms. Simulation experiments conducted show that our proposed algorithms perform well in both distance estimation and coordinate calculation, with increasing accuracy of localization of the proposed algorithms in the order M-HMM, RM-HMM, and HMM + IMM. The simulations also show that the three algorithms are accurate, stable, and robust.

## 1. Introduction

Wireless Sensor Networks (WSNs) are ideal for various scenarios, including environmental monitoring, medical care, military operations, and disaster relief [1,2,3,4,5]. In these systems, information relating the location and trajectory (*i.e*., localization) of the mobile terminals (MTs) need to be communicated. However, localization is difficult and faces a number of challenges [6]. Consequently, several methods have been proposed to solve these localization problems. The proposed methods can be broadly divided into two sets of approaches: range-free and range-based approaches. Range-free approaches attempt to localize the position of an MT without relying on the measured distances between target nodes and anchors. Instead, they calculate its position using satellites or some arithmetic according to the sequence of the signal received by the nodes. Examples of range-free approaches include global positioning system localization [7], the multiple-sequence positioning method [8], and the regulated signature distance method [9]. These methods are efficient and accurate in outdoor environments. However, they require costly hardware and are less accurate for indoor environments.

Two types of transmission are dealt with in this paper: line-of-sight (LOS) and non-line-of-sight (NLOS). If there is no obstruction to signal transmission between an MT and a base station (BS), then the transmission is said to be a LOS transmission. On the other hand, in small-scale indoor environments, obstacles such as walls, doors, metal bookcases, and even crowds, can obstruct signal propagation. In such scenarios, the transmission is said to be NLOS transmission. Range-based solutions are more suitable for this kind of environment than range-free solutions. In practical environments, NLOS and LOS transmissions are mixed.

Several range-based techniques have been proposed to reduce the effect of significant NLOS measurement errors. Range-based solutions such as the time of arrival method, time difference of arrival method, and received signal strength indicator method are commonly used to resolve localization issues [10]. On the basis of measurements obtained from these methods, a variety of mathematical techniques can be used to solve the problems, especially for the NLOS scenario. Kalman filter [11] works well for linear systems with a Gaussian assumption, whereas for nonlinear systems with a non-Gaussian assumption, the extended Kalman filter (EKF) [12], the H-infinity filter [13], and particle filters (PFs) [14] have been proposed. Of these, PF is a class of recursive Bayesian estimation filters based on sequential Monte Carlo methods. This method divides the area into several grids to form a particle of sufficient density, prior to localization. PF has been proved efficient for models of nonlinear systems and outperforms common nonlinear filters. However, the performance of PF relies heavily on the number of particles and the sequential resampling method. Furthermore, the time required for calculations is inversely proportional to the number of particles selected. Vera *et al*. presented the Easy to Deploy Indoor Positioning System [15], which is able to support the typical localization requirements involved in loosely couple mobile work base on a WIFI system. This method is aimed for fast deployment and real-time operations rather than for location accuracy.

The hidden Markov model (HMM) filter, used extensively in speech processing, is another grid-based method that utilizes Bayesian techniques to estimate the location. In HMM-based localization, a common approach is to use the Viterbi algorithm to calculate position. Morelli *et al*. [16] proposed a Detection/Tracking Algorithm (D/TA) based on this technique and reported satisfactory experimental results. Chen *et al*. [17] proposed an interacting multiple model (IMM) that combines various methods, on the basis of the probability of interaction, to solve the localization problem with satisfactory precision. Performance analysis methods for WSN localization have also been extensively researched. Of these, the Cramér–Rao lower bound (CRLB) is an optimality criterion for the simulation environment.

In this paper, we propose a method that enhances the HMM filter using a modified hidden Markov model (M-HMM). This method determines a compromise solution to improve both efficiency and accuracy. The IMM technique is then used to transform the hidden states between the high-speed and low-speed situations. Thus, it can satisfactorily simulate a real movement environment. Moreover, the IMM is treated as a two-state Markov process to interact with high-velocity and low-velocity models. The CRLB of the environmental simulation is also calculated to determine the accuracy of the algorithm. Simulation results show that our proposed method is closer to the CRLB and superior to conventional methods.

The remainder of this paper is organized as follows: Section 2 provides a brief overview of the methods that have been proposed for the elimination of NLOS errors. Section 3 discusses background assumptions made. Section 4 presents the details of the proposed modified HMM method. Section 5 presents the integrated algorithm formed by combining IMM and HMM. Section 6 presents the CRLB of the environment. Section 7 outlines the simulation experiment conducted and discusses the results obtained. Finally, Section 8 concludes this paper.

## 2. Related Work

NLOS identification and mitigation techniques have been extensively researched. Several algorithms that operate by identifying and rejecting data received in NLOS situations and using access points (APs) to calculate the position of an MT in LOS situations have been proposed to solve the NLOS localization problem. Chan *et al*. [18] proposed a method that uses a residual test to identify APs in LOS scenarios and then uses the APs identified to locate the position. Heidari *et al*. [19] proposed definitions for under-detected direct path conditions and direct path conditions, followed by a consequent identification technique that uses binary hypothesis testing and a neural network architecture. Yu *et al*. [20] proposed conducting a hypothesis testing analysis in NLOS environments, which significantly improved the accuracy of position calculation. For unknown parameters in the NLOS error method, Chen [21] used a residual weighting algorithm to mitigate the effects of NLOS error. Marano *et al*. [22,23] used a support vector machine to solve the problem of nonparametric NLOS identification. On the one hand, this method imposes a formidable computational burden during LOS selection, while on the other hand, it abandons information obtained from the APs in NLOS transmissions. Wang *et al*. [24] presented a data association scheme that incorporates LOS and NLOS range measurement into the PF framework to effect location estimation.

The localization performance of algorithms depends on the NLOS model used. Most NLOS algorithms assume that NLOS error takes the form of a Gaussian distribution. However, in real environments, the distribution of NLOS error is uncertain. Merino [25] and Wang [26] used the Gaussian Mixtures Model to solve the problem. McGuire *et al*. [27] proposed a nonparametric kernel method to calculate the propagation delay. Morelli *et al*. [16] proposed an HMM-based method that relies on high-resolution ultra-wideband (UWB) technology. This grid-based approach was proposed to jointly track the sequence of the positions and sight conditions of the MT. The HMM method does not rely on linearization and the Gaussian assumption, which is the hypothesis regarding noise background in most algorithms. Furthermore, in simulations, an exponential distribution is assumed for NLOS. Considering the large computational burden of the HMM algorithm, Nicoli *et al*. proposed a jump Markov particle filter approach to locate the positions [28]. This method is more efficient than that of Morelli *et al*. [16] while exhibiting a similar accuracy to it. In this paper, we propose a modified localization algorithm based on the HMM method that can utilize more information than that of Morelli *et al*. [16] and Nicoli *et al*. [28] regarding the signal received.

Although the above algorithms all exhibit robustness, each algorithm has another specific advantage in particular conditions. Consequently, several of these algorithms have been combined into dynamic systems using IMM. Liao *et al*. [29] proposed a Kalman-based IMM smoother that fuses LOS and NLOS conditions in cellular networks based on TOA measurements. Subsequently, Chen *et al*. [17] proposed an extended Kalman-based interacting multiple mode (EK-IMM) smoother and a fuzzy-based interacting multiple mode smoother [30] for mobile localization in order to estimate LOS/NLOS transition based on data fusion with TOA and received signal strength (RSS) measurement data. However, they assumed the mobile terminal to have a constant velocity in both methods, which affects the adaptability of the algorithm. Hammes *et al*. [31] combined the EKF in LOS and the robust EKF in NLOS with IMM. Cheng *et al*. [13] integrated the Kalman filter with the H-infinity filter in IMM to improve range measurement. Compared with the algorithm proposed by Hammes *et al*. [31], Cheng used a different arithmetic to solve problems in different situations.

In the methods proposed above, IMM is used extensively to integrate the LOS and NLOS states. However, both the LOS and NLOS information are already considered in the HMM localization model in this paper. Consequently, there is no need to switch modes between LOS and NLOS. Furthermore, the static speed model is a weakness of the arithmetic in the HMM model. Consideration of the velocity of the MT is restricted in the indoor environment, and thus we simply divide the speed model into two parts, using the IMM to render the algorithm more robust against random movements.

Several methods have been proposed to analyze the advantages and disadvantages of various algorithms in this context. They include geometric dilution of precision and the CRLB. Qi [32], Huang [33], and Yin [34] analyzed the CRLB in varying noise backgrounds and sight situations. In this paper, we use the method proposed by Huang *et al*. [33] to calculate the CRLB value of our simulated environment.

## 3. Background Assumptions

**Figure 1:**
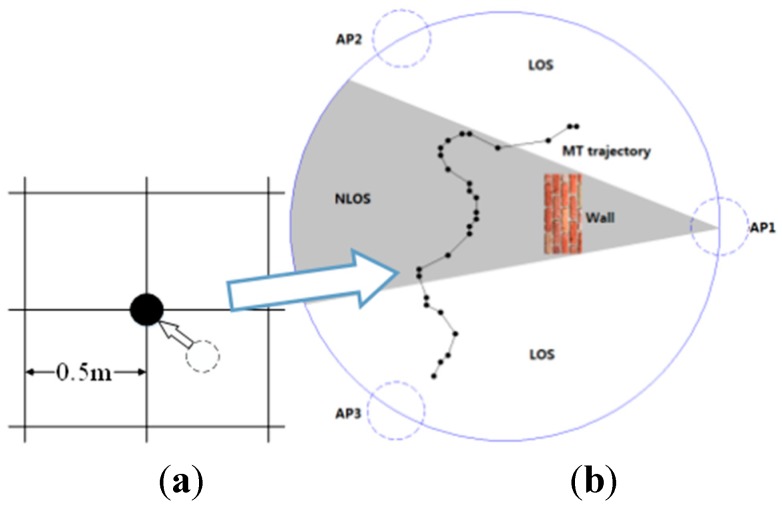
(**a**) Discrete approximate schematic diagram; (**b**) NLOS/LOS condition schematic diagram.

A virtual circular area is first hypothesized as shown in Figure 1. The MT is then assumed to carry out *k* random motions around the particular UWB infrastructure *Q* which is defined as the circumscribed circle of three static APs. Its *k*th localization position $M=\{q^{\left(i\right)},s_{j}^{\left(i\right)},j\in \{1,2,3\}\}$ is calculated by using the signal received from the three APs (j ∈ [1, 2, 3]), where $q^{\left(i\right)}=\left[x^{\left(i\right)},\;y^{\left(i\right)}\right]$ denotes the Cartesian coordinate of the MT in two-dimensional space at the *k*th time step. In HMM framework, it is impossible to get the exact sequential Bayesian inference. And as a result, all trajectory points mentioned in this paper are approximated to the nearest grid point that divides the region b, Δ*d* = 0.5 m as shown in Figure 1a. The APs are also assumed to be located on the stationary points, which trisects the circle. Further, if the *l*th AP and the MT can communicate without obstructions, as shown by the area in white in Figure 1b, this situation is defined as an LOS condition and *s_l_*^(*i*)^ = 0. Conversely, if the path between the AP and the MT is impeded by a thick wall, a metal door, or any other obstacle such that they cannot directly communicate, this situation is known as an NLOS condition, and *s_l_*^(*i*)^ is assigned a value of one in this case. To simplify this analysis, we assume that each sight condition *s_l_*^(*i*)^ is independent of position *q*^(*i*)^ and is only related to *s_l_*^(*i-*l)^. The MT can obtain signal sequence *y_i,l_* from any AP at any time.

Given the above hypothesis, the MT can be localized using knowledge of HMM probability. In the next section, we introduce a signal model and the relationship between the signal model and location probability.

According to Heidari *et al*. [19], the ideal discrete-time description equation for the indoor channel profile is characterized by:

(1) $$h(t)=\sum_{k=1}^{N_{p}}\alpha_{k}e(t-\tau_{k})$$

where *e*(⋅) represents the time-domain pulse shape of the filter, *Np* is the number of multipath components, $\alpha_{k}=\left|\alpha_{k}\right|\exp(j\phi_{k})$ represents uncorrelated fading amplitudes, θ represents the phase of the *k*th path, and τ*_k_*, including [0,τ_1_,τ_2_…τ_*k*-1_], is the time delay. We are only concerned with the first arrival delay τ_1_, for localization, which is equal to the propagation time given by:

(2) $$\tau_{1}=\;\tau \;+\;\Delta \tau \times {s_{l}}^{\left(i\right)}$$

where *s_l_*^(*i*)^ = 1. for NLOS and *s_l_*^(*i*)^ = 0 for LOS. According to Morelli *et al*., [35] τ = *d* / ( *c*Δ*t*) at any discrete-time τ, and the *l*th MT–AP link is defined as two independent real-value zero-mean white Gaussian signals:

(3) $${y_{i,l}}^{n}\left(t\right)\;=\;{z_{i,l}}^{n}\left(t\right)\;+\;{w_{i,l}}^{n}\left(t\right)$$

where ${z_{i,l}}^{n}\left(t\right)=b^{\left(n\right)}\times g\left(t\right)\;\times h^{\left(n\right)}\left(t\right),\;b^{\left(n\right)}\epsilon \;\left\{-1,\;+1\right\}$ and $g\left(t\right)=\left[1-{\left(t/Tg\right)}^{2}\right]\exp\left[-\left(1/2\right)\left({\left(t/Tg\right)}^{2}\right)\right]$, which is a second-order Gaussian pulse for user *n.* To simplify this, we consider only a single-user situation. Morelli *et al*. [16] state that *z* ~ *N*(0, *C_z_* (τ, Δτ)) is a zero-mean Gaussian vector with covariance matrix *C_z_* (τ, Δτ). The entire signal *y_i,l_* (*t*) is *N*(0, *C* (τ, Δτ)) with covariance matrix $C\left(\;\tau ,\;\Delta \tau \right)=\;I_{P}{\sigma_{0}}^{2}+C_{z}\left(\;\tau ,\;\Delta \tau \right)$, where σ_0_ is the covariance of the background noise. Moreover *C_z_* (τ, Δτ), is a diagonal matrix with diag $\left(L_{z}\left(\;\tau ,\;\Delta \tau ,\;1\right),\;\ldots ,\;L_{z}\left(\;\tau ,\;\Delta \tau ,\;P\right)\right)$:

(4) $$L_{z}\left(\;\tau ,\;\Delta \tau ,\;P\right)=\;{\sigma_{z}}^{2}\left(\tau \right)\rho^{k-\tau }u\left(p-\tau \left(1\right)\right)$$

where ρ is the attenuation factor, fixed at 0.9 in this paper, and the receiving power σ_*z*_ decreases with increasing propagation distance *d*. Note that $\;{\sigma_{z}}^{2}={\sigma_{ref}}^{2}{\left(d/\;d_{ref}\right)}^{\left(-\alpha \right)}$.

**Figure 2:**
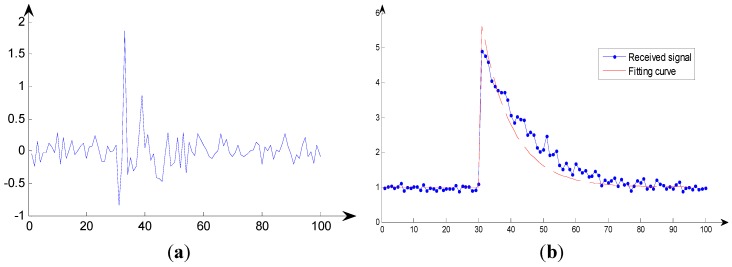

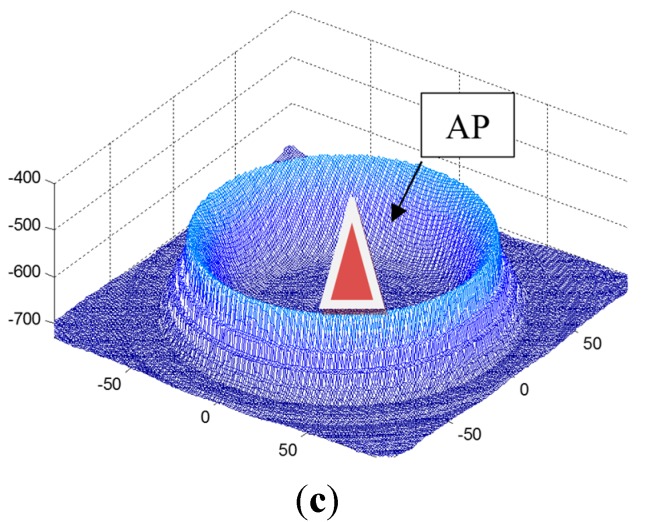
An instance of the value of the RSS power measured. (**a**) Example of receive signal; (**b**) RSS power delay profile model; (**c**) Example of log-likelihood function for the signal measured by AP.

An instance of the value of the measured RSS power is shown in Figure 2. In the Figure 2, $\tau =20,\;\Delta \tau =\;10,\;{s_{l}}^{\left(i\right)}=1$. Figure 2a shows the signal received by the AP at *P* disperse times. And in Figure 2b the solid blue line represents the absolute value of the received signal, and the red dotted line represents the fitting curve of the covariance at each time point. Figure 2c shows when an AP receives a set of signals, it can estimate the probability of the position of the source by rotating the power delay profile model. In the simulation for HMM, the NLOS delay has an exponential probability density function (PDF) ${\sigma_{\delta }}^{-1}\exp(-\tau /\sigma_{\delta })$ with σ_δ_ = 10. In this paper, we assume that the NLOS delay is generated by σ_δ_ = 7. According to the nonparametric kernel method proposed by McGuire *et al*. [27], the estimated PDF of NLOS delay is as follows:

(5) $$f(b)=\frac{1}{\sqrt{2\pi }Ph_{ij}}\sum_{t=1}^{P}\exp\left(-\frac{{\left(b-Sb_{ijt}\right)}^{2}}{2h_{ij}^{2}}\right)$$

**Figure 3:**
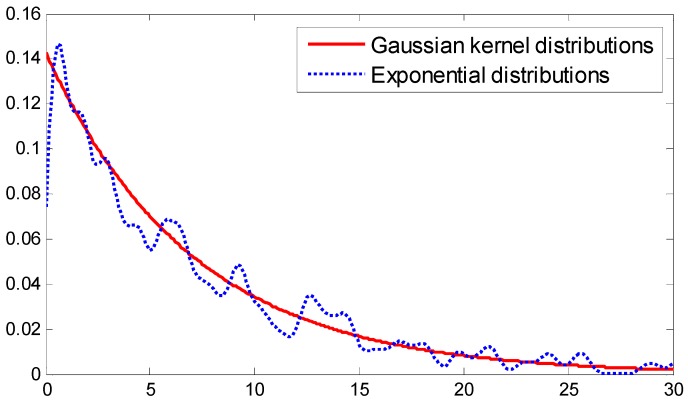
PDF comparison for Gaussian kernel distributions and exponential distributions.

In the above equation, exp(⋅) is a Gaussian kernel function, and *h_ij_* is the smoothing constant that determines the width of the kernel function. In this paper, we choose *h_ij_* as 0.4 and *P* as 200 (*cf.* Appendix A). The fitting curve is shown in Figure 3, with the red line representing the PDF of the Gaussian kernel function and the blue line denoting the PDF of the exponential distribution.

In traditional localization systems, the basic approach is to estimate the position of the MT through maximum likelihood estimators. As mentioned earlier, the maximum likelihood probability density function of the RSS model, which is the Gaussian probability density function with variables in vector form, is as follows:

(6) $$P\;\left(y_{i,l}/M^{i}\right)=\;{\left|C\left(\tau ,\;\Delta \tau \right)\right|}^{-1/2}{\left(2\pi \right)}^{-P/2}\exp\left[-1/2Y^{T}C^{-1}\left(\tau ,\;\Delta \tau \right)Y\right]$$

As long as MT obtains received signal sequence *Y* from the AP, it can calculate the distance probability. On obtaining the distance information from three non-collinear APs, we can calculate the coordinates by executing trilateration or maximum likelihood estimation by merging:

(7) $$P\;\left(Y^{i}|\;M^{\left(i\right)}\right)=\prod_{l=1:3}P\;\left(y_{i,l}|\;M^{\left(i\right)}\right)$$

Although this method works well for the LOS case, it has shortcomings. On the one hand, when the MT and AP are in an NLOS situation, significant errors can occur. Further, this method does not consider the regular pattern of motion of the MT, in which the probability of the movement at any moment contains information related to the signal sequence. The HMM algorithm and our proposed improvement on it, described in the next section, solve these problems.

## 4. Localization Based On HMM

An HMM is a statistical Markov model in which the system modeled is assumed to be a Markov process with unobserved latent variables. On the basis of the mathematical models used in this article, location variable $M_{i}=\;\left[q^{\left(i\right)},{s_{l}}^{\left(i\right)}\right]$ is not directly visible, but output *Y^i^*, depending on the state, is perceptible. Each state *M_i_* has a probability distribution over possible output *Y^i^*. Therefore, sequence *Y^i^* expresses some information about the sequence of the location states. To solve the classical HMM problem, we need to construct a transition probability matrix *A* = {*P*(*M_i_*│*M_i_*_‒1_)}, an observation probability matrix *B* = {*P*(*Y_i_*│*M_i_*_‒1_)}, and an initial value π = {*P*(*x*_0_)}. In this paper, we initially set *P*(*x*_0_) = (5,7),(000), which means that the initial moving point is at (5, 7) and the MT is in an LOS condition for all three APs.

### 4.1. Matrix Based on Dynamic State

As $M_{i}=\;\left[q^{\left(i\right)},{s_{l}}^{\left(i\right)}\right]$, the transition probability *P* (*M_i_*│*M_i_*_‒1_) can be divided into two parts: position transition probability (PTP) and sight transition probability (STP):

(8) $$p\;\left(M_{i}|M_{i-1}\right)=p\left(q^{\left(i\right)}|q^{\left(i-1\right)}\right)\times p\left({s_{l}}^{\left(i\right)}|{s_{l}}^{\left(i-1\right)}\right)$$

In the real environment, STP is related to position information. In brief, it assumes that the probability of the sight state maintaining its status is 0.7 compared with the previous moment. Consequently, the probability of a change in the sight state is 0.3.

In this paper, we focus on the calculation of PTP. Morelli *et al*. [16] described this in three forms, with the circular Gaussian PDF scenario simulated as a sample. However, they consider neither the actual trajectory of the MT nor inertia in the direction of motion. In order to solve this problem, Ru *et al*. [36] changed the weights of different angles based on the laws of motion obeyed by objects in different situations, which is a very efficient method. The formulation then changes as follows:

(9) $$p\;\left(q^{\left(i\right)}|q^{\left(i-1\right)}\right)=\frac{1}{\sqrt{2\pi }\sigma }e^{\frac{-d}{2\sigma^{2}}}f(\Delta Q,d)$$

**Figure 4:**
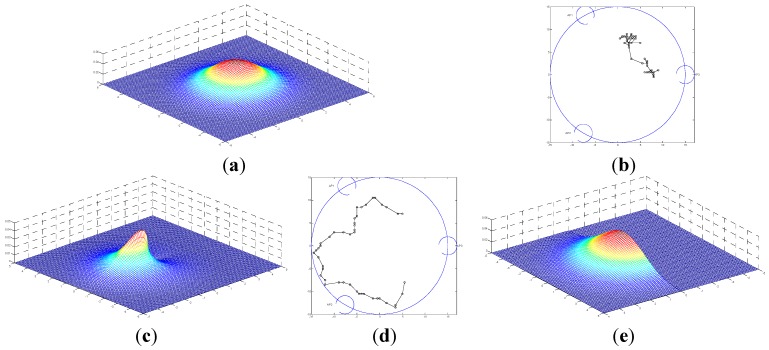
Examples of *P*(*m^i^*│*m^i^*^‒1^) PDF models. The arrows represent the forward direction of the MT: (**a**) Circular Gaussian PDF with deviation σ*_v_* = 3 in [16]; (**b**) Trajectory of (**a**). (**c**) Gaussian distribution with variable ΔQ and σ = 0.5; (**d**) Trajectory of (c); (**e**) PDF with ΔQ ϵ [−90°, 90°].

Here, f(ΔQ,d) is the additional weight with two parameters, namely, the distance *d* between *M_i_*_-1_ and *M_i_*, and the deflection angle ΔQ between *M_i_*_-1_ and *M_i_*_-2_. If the MT moves as a circular Gaussian PDF with deviation σ = 3, as shown in Figure 4a, where the MT moves at any angle with the additional weight one, the trajectory of the simulation for 50 steps is shown in Figure 4b. Similarly, if the additional weight is a Gaussian function with variable ΔQ, the trajectory is as shown in Figure 4d. Thus, the trajectory changes as the model changes, which also implies that different sorts of motion correspond to different models. Moreover, it is clear that the more similar the mathematical model is to the phenomena, the more accurate will be the results obtained. Thus, it is not suitable to use separate PTPs for each motion model. Consequently, we introduce an interacting multiple model algorithm in the ensuing sections to solve this problem.

### 4.2. Detection/Tracking HMM

In this section, we look at a more efficient HMM. In HMM theory, there are two common methods for extracting potential relationships between the signal sequence *Y* and the state *M*. The backward/forward algorithm (BFA) is first invariably used to estimate the probability of an observed sequence provided by an HMM. The Viterbi algorithm (VA) provides a similar method to calculate the most probable sequence by traversing all *P*(*m*^0:(*i-*1)^│*y*^0:(*i*-1)^) and determining the most efficient sequence, *m*^0:*i*‒1^. However, neither BFA nor VA is appropriate for the localization problem [16]. The Detection/Tracking Algorithm (D/TA), which uses the HMM to satisfactorily solve the localization problem, was subsequently proposed by Spagnolini and Ram [37] and employed to estimate the localization by Morelli *et al*. [16]. The main idea underlying the D/TA is to find the maximum value of *P*(*m^i^*│*y*^0:*i*^) in all signals received at the *i*th moment, which is quite similar to the process of calculating *P*(*m^i^*│*y*^0:(*i*-1)^) in the ‘forward’ step of the BFA. Its fundamental formula is as follows:

(10) $$P(m^{i}|y^{0:i})=p(y^{i}|m^{i})\sum_{m^{i-1}\in M'}p(m^{i}|m^{i-1})P(m^{i-1}|y^{0:(i-1)})$$

The flow diagram below explicates the working principle of the D/TA in HMM.

**Figure 5:**
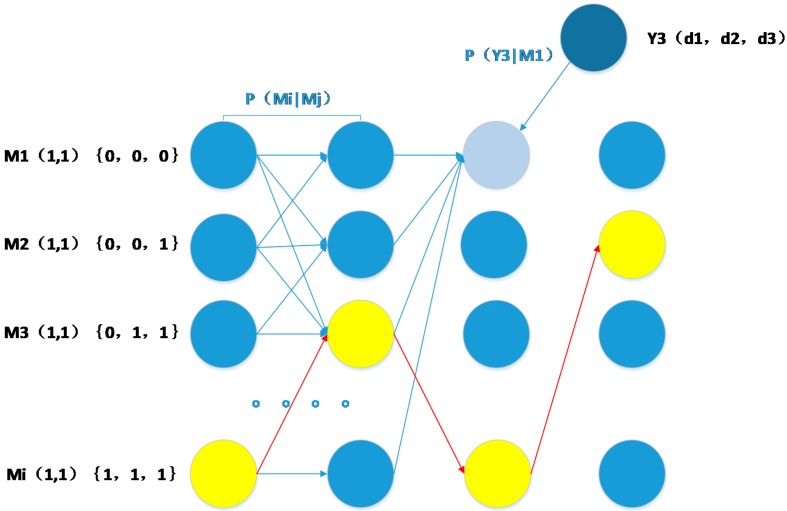
Flow diagram of D/TA.

**Figure 6:**
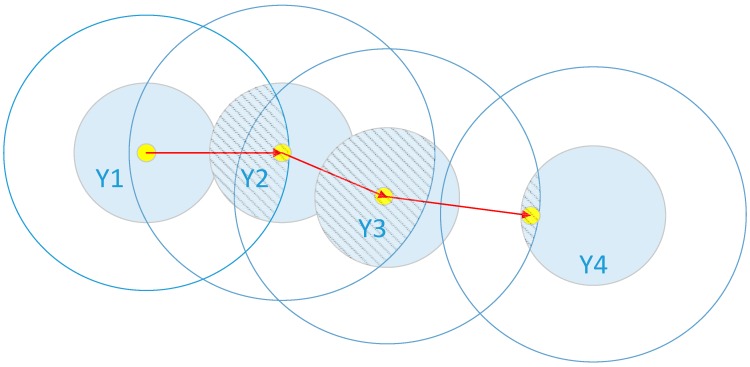
Flow diagram of the location algorithm.

In Figure 5, each node represents a probability calculated for each state. Each line represents a different state, as stated above, and the values of each column change with time. *Y_i_* represents the signal received at the corresponding time *i*. For example, the node in the third column and first line represents the probability that the state is M1(1, 1){0, 0, 0}, which means that the coordinates of the MT are (1, 1) and that the MT is in LOS with AP1, AP2, and AP3 at *i* = 3, with the signal received Y3(*d*1, *d*2, *d*3) represented by the blue-black circle. Each arrow maps the multiplication state *M_i_* to the transition probability *P*(*M_i_*│*M_i_*_‒1_), and the line linking them signifies a summation operation. We calculate the probability *P*(*m^i^*│*y*^0:1^) of every state at time *i* based on the result calculated in the last step. Finally, the maximum of each line is extracted and the final trajectory is determined corresponding to the coordinates obtained. By integrating the flow diagram with the formula, the entire process of positioning can be described as follows: in Figure 6, the yellow points represent the estimated location for the MT and the circular ring represents the area that the MT might occupy the next moment. Note that although the MT can be anywhere the next moment, we ignore positions where the MT might occur with a probability that has empirically been determined to be negligibly small.

In Figure 6, the transition probability *P*(*M_i_*│*M_i_*_‒1_) is selected as a function distributed uniformly to simplify the analysis. The blue circles represent areas that can receive signals at any given time. The detailed procedure for localization is as follows:

a. Confirm an initial value, or the corresponding values of the previous time point.
b. Determine where the MT will be and draw a hollow ring depending on *P*(*M_i_*│*M_i_*_‒1_).
c. Use the blue circle to represent the signal source of the MT, depending on *P*(*y^i^*│*m^i^*) and the signal received at time *i*.
d. Combine Steps 2 and 3, and eliminate the area marked by the shadow.
e. Obtain the position of the MT using the weighted sum of probability *P*(*m^i^*│*y*^0:1^) and the coordinates. Then, mark a yellow dot and link it to the previous point, shown by the red line in Figure 6.

This method is limited in that it only considers past and present situations but loses sight of the “future.” In Section 4.3, we present a novel future-based modified method that solves the localization problem from a broader perspective. From another standpoint, it can be considered as a correction for past records.

### 4.3. Modified HMM

**Figure 7:**
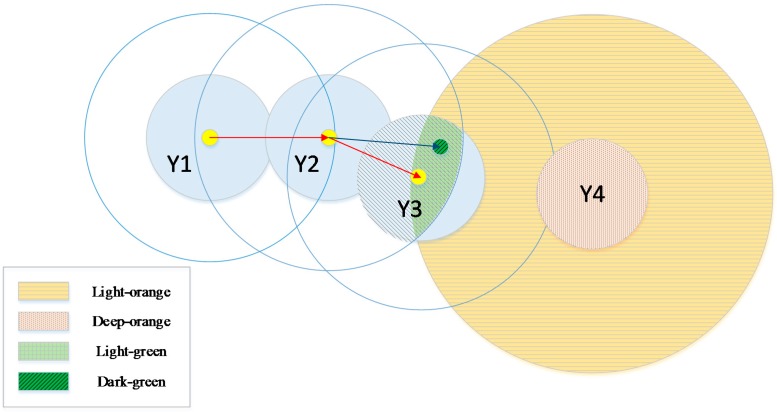
Flow diagram of HMM location algorithm.

The location is calculated only once in ordinary localization theory for each position. There is no modification from the next time, *i +* 1. In other words, if we once fix the position at the *i*th moment in time, the accuracy of this determination will not be considered at time *i +* 1 because it reduces efficiency. However, the position in the *i* + 1th moment and the signal received in the *i* + 1th moment are important for estimation of the position in the *i*th moment. Because most signals are reliable and the signal at the next moment in time usually contains hidden information regarding the location of the MT, the weight of the state estimated at the preceding moment in time should be reassigned based on transition probabilities *P*(*M_i+1_*│*M_i_*) and observation probabilities *P*(*y^i^*^+1^│*m^i^*^+1^). In other words, the weight of the state with a greater probability of receiving the signal should have more merit, and this corresponds to the value of *P*(*m^i^*│*y*^0:(*i*+1)^) . Through this method, we solve the smoothing problem in a discrete way HMM to improve the localization precision. To explicate this idea, another schematic diagram is shown in Figure 7.

In Figure 7, the meaning of the pattern is virtually identical to that in Figure 6, except for the parts in orange and green. The deep-orange solid circles signify areas that can receive signal *Y*4 at the fourth time step and the light-orange solid circles signify areas where the MT might be located at the third time step. The precondition for these is based on the transition probabilities *P*(*M_i+1_*│*M_i_*) and signal *Y*4. In short, the light-orange area constitutes a new limited condition on the MT at the third time step. It shows where the MT could come from. Using this scope, the boundary of the MT estimated at the third time step is further reduced, which is shown as the light-green area in the figure. The yellow point estimated at the third time step is then transferred to the dark-green one, as the diagram shows. Steps *a* to *e* of localization are identical to those listed in Section 4.2. The following are the additional steps:

f. Receive the signal in the “future” *Y^i^*^+1^.
g. Calculate area *Y^i^*^+1^ based on *P*(*y^i+1^*│*m ^i^*^+1^) , and represented by the deep-orange solid circle.
h. Calculate the limiting condition based on both *P*(*M_i+1_*│*M_i_*) and the area obtained in Step 7, and mark it with light-orange.
i. Shrink the possible area shown around *Y*3 from Step 8 using the area shadowed in Figure 5 around *Y*3 from Step 4. Obtain a new scope filled with light-green.
j. Perform the same calculations as in Step 5 using the weighted sum of probability *P*(*m^i^*│*y*^0:(*i*+1)^) and the coordinates. Finally, mark the location as a dark-green point and link it to the last point, which is shown as the blue line in Figure 6.

The traditional localization methods mentioned above are used to calculate the probability *P*(*m^i^*│*y*^0:*i*^) or *P*(*m^i^*│*y*^0:(*i*-1)^). These methods always take into account efficiency and precision, and the method presented in this subsection is intermediate. Having outlined the principle of the modified method, we can represent it as follows:

(11) $$P(m^{i}|y^{0:i+1})=P(m^{i}|y^{0:i})\sum_{m^{i+1}\in M'}p(m^{i+1}|m^{i})p(y^{i+1}|m^{i+1})$$

**Figure 8:**
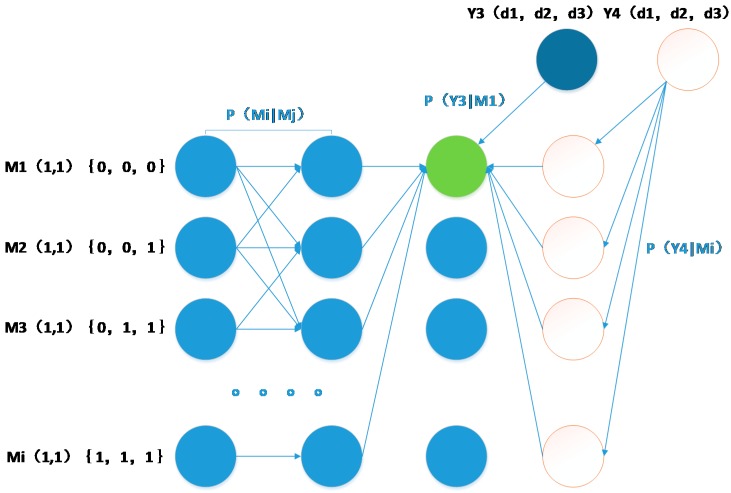
Flow diagram of the M-HMM algorithm.

A flow diagram that calculates *P*(*m*^1^│*y*^0:4^) is shown in Figure 8.

In the figure, we can distinguish the proposed method from the one mentioned above. The legends are similar to the ones in Figure 6, with the difference that we here need to reflect on backward multiplication based on received signal *y*^4^, the observation probability *P*(*y*^4^│*m*^1^), and the transition probability at each state. From the formula and the figure above, we know that probability *P*(*m^i^*│*y*^0:*i*^) is the basis for probability *P*(*m^i^*│*y*^0:(*i*+1)^), and thus this method involves an unavoidable delay. In real-time processing, we should calculate the coordinate information *P*(*m^i^*│*y*^0:*i*^) by D/TA first and display it to users temporarily. Then, the M-HMM method should be used to modify the calculated position. The performance is enhanced because of the readjustment calculation of every unknown coordinate in the trajectory that provides a balance between precision and efficiency. 

### 4.4. Replacement of the Modified HMM Method

In the previous subsection, we proposed a modified HMM method to improve localization accuracy. The fundamental part of the modified algorithm is the calculation of *P*(*m^i^*│*y*^0:(*i*+1)^) at each *i +* 1 moment. However, as the results of our simulation subsequently show, *P*(*m^i^*│*y*^0:(*i*+1)^) exhibits better performance than traditional *P*(*m^i^*│*y*^0:*i*^) estimation. Given the above description of the HMM algorithm, the calculation process can be regarded as an iteration in which the ratio coefficients of each state *M_i_* = [*q*^(*i*)^,*s_l_*^(*i*)^] update themselves through the HMM chain. Because the trajectory obtained from *P*(*m^i^*│*y*^0:(*i*+1)^) is more accurate than that obtained from *P*(*m^i^*│*y*^0:*i*^) on the *i*th occasion, the modified ratio coefficient of each state calculated from *P*(*m^i^*│*y*^0:(*i*+1)^) may also be more accurate than the ones obtained from *P*(*m^i^*│*y*^0:*i*^). In other words, at the (*i* + 1)th time step, when we calculate state probability *P*(*m^i^*^+1^│*y*^0:(*i*+1)^), the initial iteration coefficient of *P*(*m^i^*│*y*^0:*i*^) can be replaced by *P*(*m^i^*│*y*^0:(*i*+1)^) from earlier. In actual fact, *P*(*m^i^*│*y*^0:(*i*+1)^) are different from *P*(*m^i^*│*y*^0:1^) and *P*(*m^i+1^*│*y*^0:(*i*+1)^) will also change its original meaning. Thus, we use symbols *RP*(*m^i^*│*y*^0:(*i*+1)^) and *RP*(*m^i+1^*│*y*^0:(*i*+1)^) to express them, as follows:

(12) $$RP(m^{i+1}|y^{0:(i+1)})=p(y^{i+1}|m^{i+1})\sum_{m^{i}\in M'}p(m^{i+1}|m^{i})\times RP(m^{i}|y^{0:(i+1)})$$

This replacement method can only be used when *i* ≥ 3. Furthermore, as our simulation subsequently shows, this replacement makes a slight contribution to improving localization accuracy. Although M-HMM and RM-HMM improve localization precision, this method is deficient because, in practice, the transition probability matrix should not be unique for variable motion of the MT.

## 5. Combination of HMM and IMM

In practice, the single transition probability matrix model produces numerous errors because of the variable motion of the MT. This is because each velocity distribution corresponds to a reasonable probability function. Although we can choose a uniform distribution function or other functions, these yield imprecise results. The best approach to solve this problem is to create several separate transition probability models and derive results depending on the velocity distribution in each. The IMM estimator is one of the most effective approaches to this problem in uncertain environmental conditions, where a dynamic system with multiple switching probabilities is used to select the proper transition probability model at the appropriate time.

**Figure 9:**
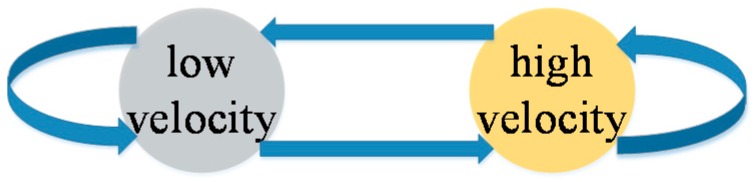
The two models proposed in this paper.

The MTs in this paper refer to robots or human, and their velocities usually range from 0 to 4 m/s. We thus divide the velocities into low- and high-velocity parts in order to simplify our model. The low-velocity part is a Gaussian function with mean value zero and variance 1.5, and the high-velocity part is one with mean value three and variance 1.5. Note that the narrower the spaces into which we divide the velocity, the more precise the results we obtain. However, our two-part strategy is effective in describing the trajectory of the terminal in this narrow range of velocities. Figure 9 shows the Markov switching model, which shows that the system varies between the low- and high-velocity models, where *P_ab_* represents the Markov transition probability from mode a to mode b.

**Figure 10:**
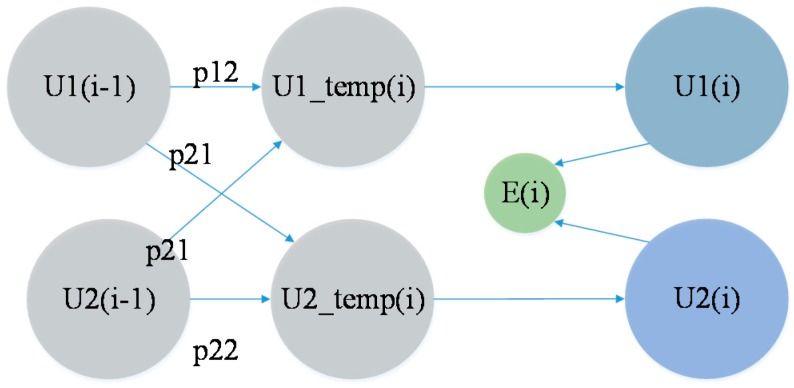
Flow diagram of the IMM algorithm.

A schematic diagram of IMM is shown in Figure 10.

In the figure, U_1_(i−1) represents the probability of the presence of the MT in the low-velocity model at time i−1, U_2_(i−1) represents the probability of the presence of the MT in the high-velocity model at time i−1, and E(*i*) represents the position estimated at time *i,* which is the final position. From the figure, we know that the system selects a model based on $\sum_{m\in M}P_{1}(m^{i}|y^{0:(i+1)})$ and$\sum_{m\in M}P_{2}(m^{i}|y^{0:(i+1)})$, which are considered the prior probabilities of each model. It then adds them to the Markov chain and calculates the weight of each model at the final position. The system subsequently calculates the coordinates based on the weighted sum of U_1_(*i*) and U_2_(*i*) along with their coordinates *l*_1_(*i*) and *l*_2_(*i*), respectively. This process can be expressed by the following equations:

(13) $$U_{1}(i)=\sum_{j}(1/C_{1})\cdot P_{j1}\cdot U_{j}(i-1)\cdot \sum_{m\in M}P_{1}(m^{i}|y^{0:(i+1)})$$

(14) $$U_{2}(i)=\sum_{j}(1/C_{2})\cdot P_{j2}\cdot U_{j}(i-1)\cdot \sum_{m\in M}P_{2}(m^{i}|y^{0:(i+1)})$$

(15) $$C_{1}=\sum_{j}P_{j1}\cdot U_{j}(i-1)\cdot \sum_{m\in M}P_{1}(m^{i}|y^{0:(i+1)})$$

(16) $$C_{2}=\sum_{j}P_{j2}\cdot U_{j}(i-1)\cdot \sum_{m\in M}P_{2}(m^{i}|y^{0:(i+1)})$$

(17) $${q_{1}}^{\left(i\right)}=\sum_{l\in Q}P_{1}(m^{i}|y^{0:(i+1)})\cdot l_{i}$$

(18) $${q_{2}}^{\left(i\right)}=\sum_{l\in Q}P_{2}(m^{i}|y^{0:(i+1)})\cdot l_{i}$$

(19) $$E(i)=\frac{U_{1}(i)\cdot {q_{1}}^{\left(i\right)}+U_{2}(i)\cdot {q_{2}}^{\left(i\right)}}{U_{1}(i)+U_{2}(i)}$$

**Figure 11:**
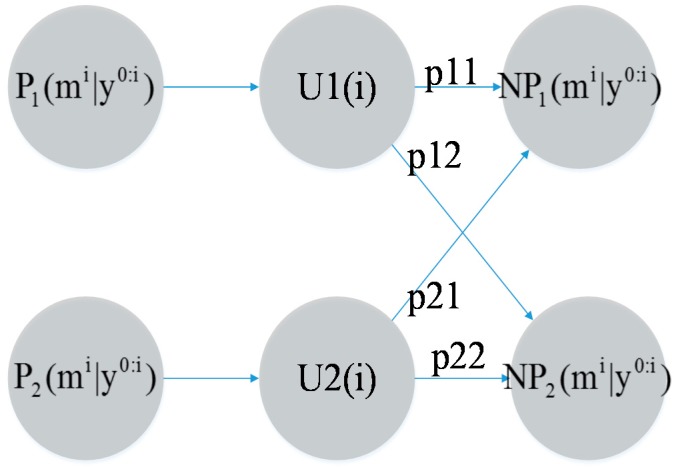
Updating of weights.

In the above equations, *C*_1_ and *C*_2_ represent the normalized coefficients of the low-velocity model and the high-velocity model, respectively; *P*_1_(*m^i^*│*y*^0:(*i*+1)^) and *P*_2_(*m^i^*│*y*^0:(*i*+1)^) express *P*(*m^i^*│*y*^0:(*i*+1)^) based on different *P*(*M_i_*│*M_i_*_‒1_) models, as described above; *l_i_* represents the coordinate information of the MT at time *I;* and *q*_1_^(*i*)^ and *q*_2_^(*i*)^ represent the positions estimated based on two sequential frames. Finally, the system updates the models and plugs the values of *NP*_1_(*m^i^*│*y*^0:*i*^) and *NP*_2_(*m^i^*│*y*^0:*i*^) into each model as the initial values for the next calculation, as illustrated in Figure 11.

The following equations express the schematic diagram:

(20) $$NP_{1}(m^{i}|y^{0:i})=\sum_{j}\left(1/C_{1})\cdot P_{j1}\cdot U_{j}(i)\cdot P_{j}(m^{i}|y^{0:i}\right)$$

(21) $$NP_{2}(m^{i}|y^{0:i})=\sum_{j}\left(1/C_{2})\cdot P_{j2}\cdot U_{j}(i)\cdot P_{j}(m^{i}|y^{0:i}\right)$$

where each *NP*_1_(*m^i^*│*y*^0:*i*^) and *NP*_2_(*m^i^*│*y*^0:*i*^) is an update for a model used to continue the long-term evolution of the system. And both models are independence. In the next section, we demonstrate the feasibility of our proposed method and its superiority over other methods via simulations.

## 6. Cramér–Rao Lower Bound on Localization Error in NLOS Environments

The CRLB is a theoretical lower limit for the variance or covariance matrix of any unbiased estimate of an unknown parameter(s). The effects of position precision can be better demonstrated using CRLB, which involves using a nonparametric kernel method to build a probability density function of NLOS errors. The CRLB is also derived in NLOS. In this paper, the arithmetic introduced by Huang *et al*. [33] is used to estimate the value of CRLB related to the deployment of the APs detailed in Section II. The MT with unknown coordinates, *x*_1_*y*_1_….*x_n_y_n_*, and the APs with known coordinates, $x_{n+1}y_{n+1}...x_{n+3}y_{n+3}$, are deployed as described in Section III. The vector of the unknown parameters is $\text{θ}={\left[x_{1}...x_{n}y_{1}...y_{n}\right]}^{T}$ If $\hat{\text{θ}}$ is an estimate of θ, the CRLB of this situation can be defined as:

(22) $$E_{\text{θ}}[(\hat{\text{θ}}-\text{θ}){\left(\hat{\text{θ}}-\text{θ}\right)}^{\text{Τ}}]\geq J_{\text{θ}}^{-1}$$

where $J_{\text{θ}}^{-1}$ is the inverse of the Fisher information matrix (FIM), defined as follows:

(23) $$J_{\text{θ}}=E\left[\frac{\partial \ln f\left(r|\text{θ}\right)}{\partial \text{θ}}\cdot {\left(\frac{\partial \ln f\left(r|\text{θ}\right)}{\partial \text{θ}}\right)}^{\text{Τ}}\right]$$

where *r* represents observation matrix, *Y*. The log of the joint conditional PDF is:

(24) $$\ln f(r|\text{θ})=\sum_{i=1}^{3+n}\sum_{j<i}l_{ij}$$

(25) $$l_{ij}=\ln f_{ij}\left(r_{ij}|\left(x_{i},y_{i},x_{j},y_{j}\right)\right)$$

Considering that the area of the simulation region is 30 m × 30 m, the limited link capacity, H, is neglected. Furthermore, the positions of the APs are fixed and can be precisely obtained; thus, the information matrix corresponding to the statistics of ranging error in this environment is also neglected. The FIM in CRLB for the case without uncertainty [33] can then be written as:

(26) $$J=\left[\begin{array}{cc} J_{xx} & J_{xy}\\ J_{xy} & J_{yy} \end{array}\right]$$

Further, the CRLB can be divided into two parts, which can be written as:

(27) $$CRLB=\frac{1}{A}trace\left\{{\left({\tilde{J}}_{xx}-{\tilde{J}}_{xy}{\tilde{J}}_{yy}^{-1}{\tilde{J}}_{xy}^{T}\right)}^{-1}+{\left({\tilde{J}}_{yy}-{\tilde{J}}_{xy}^{T}{\tilde{J}}_{xx}^{-1}{\tilde{J}}_{xy}\right)}^{-1}\right\}$$

where $\left[{\tilde{J}}_{xx}\right]={\left[{\tilde{J}}_{xx}\right]}_{A=1},\left[{\tilde{J}}_{xy}\right]={\left[{\tilde{J}}_{xy}\right]}_{A=1},\left[{\tilde{J}}_{yy}\right]={\left[{\tilde{J}}_{xy}\right]}_{A=1}$.

One part of the CRLB is the parameter 1/*A*, which is a function containing information on the background noise and the NLOS errors in the environment. Moreover, parameter A can be divided into an LOS part, *A_LOS_*, and an NLOS part, *A_NLOS_*. The relationship between these two parts can be extracted as:

(28) $$A=p_{LOS}\cdot A_{LOS}+p_{NLOS}\cdot A_{NLOS}$$

(29) $$p_{LOS}+p_{NLOS}=1$$

where *p_LOS_* and *p_NLOS_* are the probabilities of the occurrence of the LOS error and the NLOS error, respectively, and should give a total sum of one. Thus, it is clear that when the NLOS error is very small, the value of *A_NLOS_* is close to that of *A_LOS_* and the total value of A is close to that of *A_LOS_*. In this paper, the value of both *p_LOS_* and *p_NLOS_* is estimated to be 0.5 for the simulation environment.

The *trace*{ } function in Equation (27) above contains information about the system’s geometric distribution. In this paper, the positions of the APs are fixed but those of the MT are randomly generated. For flexibility, we generated 10,000 rational trajectories with 80 steps and calculated their average value. The vector with the value closest to the average value was selected as the most representative trajectory vector to be used as a reference vector.

## 7. Simulations and Results

In this section, we discuss the simulations conducted to establish the effectiveness of our modified method as well as the precision attained by combining the IMM and HMM using simulation diagrams generated in MATLAB. The PF method proposed by Morelli *et al*. [35] was also used to estimate the equal trajectory in the same signal receiving framework.

We first executed a simulation in a circular environment, as described in Section II (R = 15 m), and set out three APs evenly at the edge of the area. (A robot or a human should actually walk randomly in this area with a terminal communicating with the APs.) We then generated an MT trajectory, as shown in Figure 6, using Equation (9) with ΔQ ϵ [−45°, 45°] and σ*_v_ =* 3 in 50 iterative steps. The parameters were the same as utilized by Morelli *et al*. [16]: an environment with white Gaussian noise with zero mean and variance σ_0_^2^ *=* 2, path loss with exponent α = 2.4 and ρ = 0.9 and reference distance *d_ref_* = 2. An additional NLOS Δτ was created in line with the discrete exponential PDF: σ*_d_*^‒1^exp(‒*k*/σ*_d_*), where σ*_d_* = 7. Sampling frequency *f_s_ =* 1 GHz, reference SNR η*_ref_* = 40 dB, channel delay spread τ*_rms_* = 10 ns.

This mathematical model was first used to locate the position for a constant trajectory in order to compare the ML, D/TA, PF, improved [36], and the modified and replacement algorithms proposed in this paper. The aim is to highlight the improvement in precision by using the modified method ΔQ∈[−90°, 90°] was considered to simplify the analysis and eliminate the data training process. The trajectory was generated as described before. Furthermore, it is worth mentioning that the MT rebounds back into the circle when it “impacts” the edge of the circle, just as light does.

**Figure 12:**
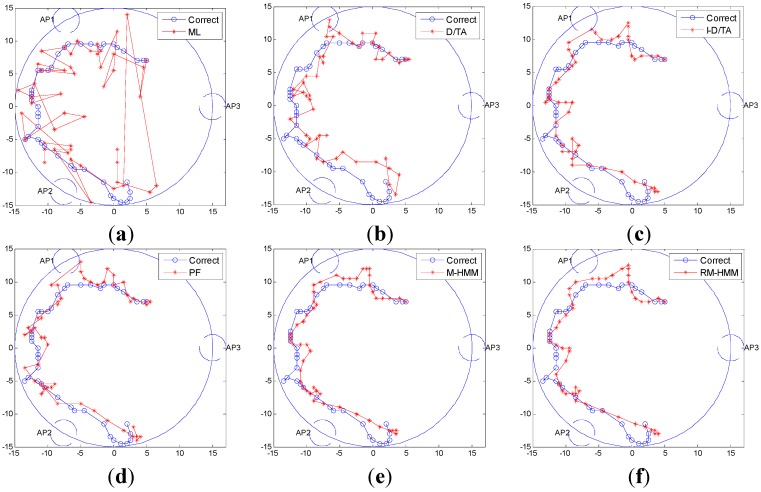
Simulation trajectory of various algorithms: (**a**) Trajectory estimated by the ML algorithm; (**b**) Trajectory estimated by the D/TA estimation in [16]; (**c**) Trajectory according to I-D/TA estimation; (**d**) Trajectory estimated by the PF algorithm; (**e**) Trajectory estimated by the M-HMM algorithm; (**f**) Trajectory using RM-HMM estimation.

The results for the six algorithms are displayed in Figure 12. Figure 12a shows the simulation based on calculations using the ML algorithm, Figure 12b shows that based on calculations using the D/TA, Figure 12c represents that using I-D/TA, Figure 12d shows the results of the simulation based on the PF algorithm, and Figure 12e,f show simulations based on our proposed modified method and replacement method, respectively.

The blue and the red trajectories in the figures refer to the correct and estimated paths, respectively. From Figure 12a, we know that the ML estimation contains several false points. Figure 12b shows that the estimated points show a large disparity with the true trajectory, especially in the determination of the tendency of the MT movement. However, no false points are apparent. This is because the improved $p\;\left(m^{i}|m^{i-1}\right)$ is closer to the real-world situation (ΔQ∈[−45°, 45°]). Figure 12c shows that the improved algorithm indeed improves the accuracy of localization, whereas Figure 12d shows that the PF algorithm is similarly accurate to the D/TA algorithm. Figure 12e shows that the trajectory calculated by the modified algorithm is smoother and more stable, and Figure 12f shows that the replacement algorithm is more accurate than the M-HMM algorithm.

**Figure 13:**
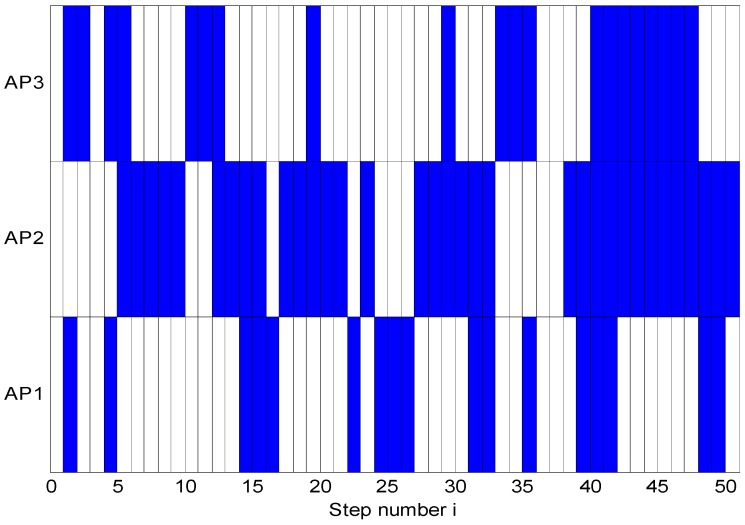
LOS/NLOS situations.

The sight conditions with respect to all APs are represented in Figure 13, where the blue and white squares represent NLOS and LOS situations, respectively. It is clear that the MT is in an adverse sight condition at the conclusion of the constant trajectory.

**Figure 14:**
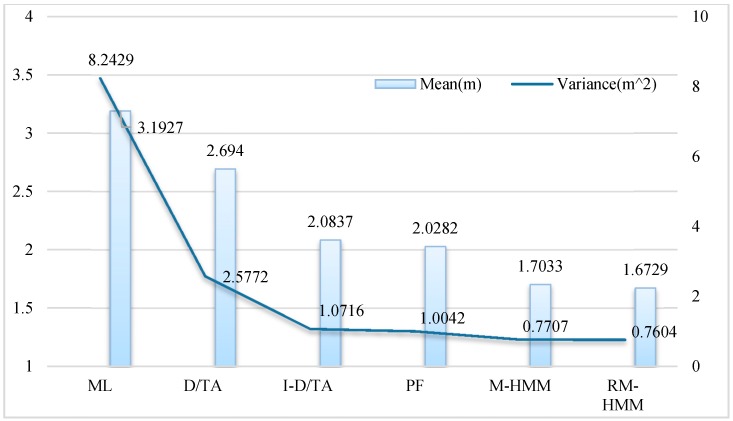
Means and variances of the six algorithms.

The average error and the variance of the six algorithms are shown in Figure 14. From the calculated data, it can be seen that I-D/TA has a much greater effect on the simulated trajectory than ML estimation, even though D/TA performs significantly better than the ML algorithm. The PF algorithm exhibits a similar accuracy and stability to that of I-DT/A. In the same manner, M-HMM and RM-HMM show similar accuracy and stability, whereas the RM-D/TA algorithm is the most accurate and stable method.

**Figure 15:**
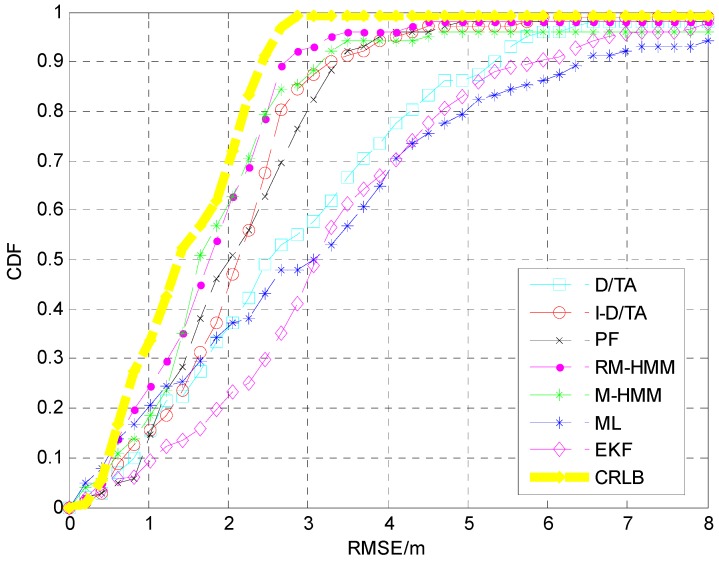
CDF analysis of the six algorithms.

In order to better represent the error, a cumulative distribution function (CDF) was plotted for the simulation, Figure 15. The blue line represents the CDF of the estimation using the ML algorithm, the cyan line represents that of the D/TA, and the red line represents that of the I-D/TA [36]. The CDF of the PF has accuracy similar to that of the I-D/TA. The RM-HMM and M-HMM also show similar precision, with the former exhibiting the best performance. In Figure 15, the accuracy of the proposed algorithm can be clearly seen.

**Figure 16:**
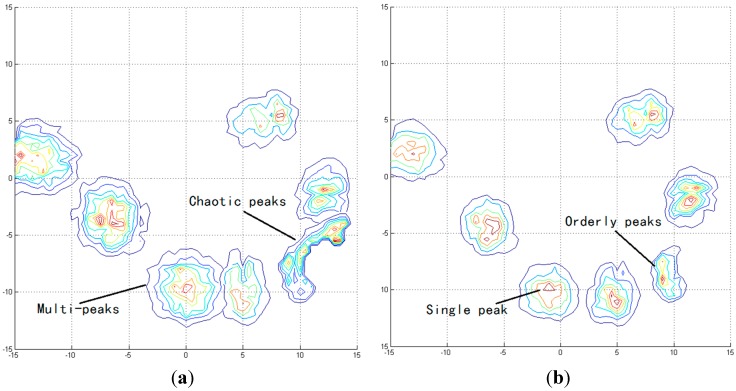
Analysis of five dispersion points based on *P*(*y^i^*│*m^i^*).

We calculated the probability *P*(*y^i^*│*m^i^*) of the fourth, 14th, 24th, 34th, 39th, and 45th steps to show the transformation from the original algorithm to the improved algorithm. To highlight the improvement, we used contours to represent the probability, with higher probability corresponding to a deeper shade of the relevant color representing it. At every step, the position of the MT was estimated by summing the coordinates and the corresponding *P*(*y^i^*│*m^i^*). Consequently, the pattern of the contours can be used to illustrate the precision of the algorithm. Figure 16a shows the original *P*(*y^i^*│*m^i^*) of the I-D/TA, whereas Figure 16b shows that of M-HMM. It can be seen that the multi-peaks and chaotic peaks obtained in the original algorithm have been reduced in number by M-HMM, which is hence more accurate.

**Figure 17:**
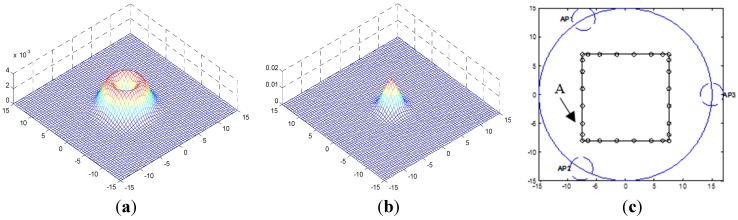
PDFs of the high- and low-speed models and the original path.

We now describe the use of simulation to establish the effectiveness of the combination of the IMM and the HMM algorithms and compare them with the methods discussed above. We first assumed the new trajectory shown in Figure 17c. In consideration of the speed of robots and humans, we assumed that the acceleration of the MT was 1 m/s^2^ with a maximum velocity of 3 m/s.

*P*(*Y^i^*│*M^(i)^*) Further, in view of the building corners, the steering angle at every corner was assumed to be a right angle, which is, in practice, a difficult situation. The MT moved around the square four times (*i* = 128). It began at (−7.5 m, −8 m), moved to the right, accelerated uniformly, slowed, and then swerved for each turn. The dispersal motion model is as follows:

(30) n=mod(i−1,8)

(31) $$V_{n}=V_{n-1}+a\cdot n\text{ (0}\leq \text{n}\leq 2\text{)}$$

(32) $$V_{n}=V_{top}\text{ (3}\leq \text{n}\leq 5\text{)}$$

(33) $$V_{n}=V_{n-1}-a\cdot n\text{ (6}\leq \text{n}\leq 7\text{)}$$

where *i* represents discrete time and *V_top_* = 3 m/s, a = 1 m/s^2^, *V*_0_ = 0 m/s, and *n* is iterated from zero to seven. A value of n = 0 signifies that the MT is at a bend or turn; in this case, *V_n_* is 0 m/s, which means that the MT stops once at every corner. The MT iterated 17 times through the trajectory. The high-speed model was chosen to be a Gaussian function with an average value of three and a variance of 1.5, as shown in Figure 17a. The low-speed model was selected to be a Gaussian function with an average value of zero and variance of 1.5, as shown in Figure 17b. The values of the parameters of the model were empirically determined.

**Figure 18:**
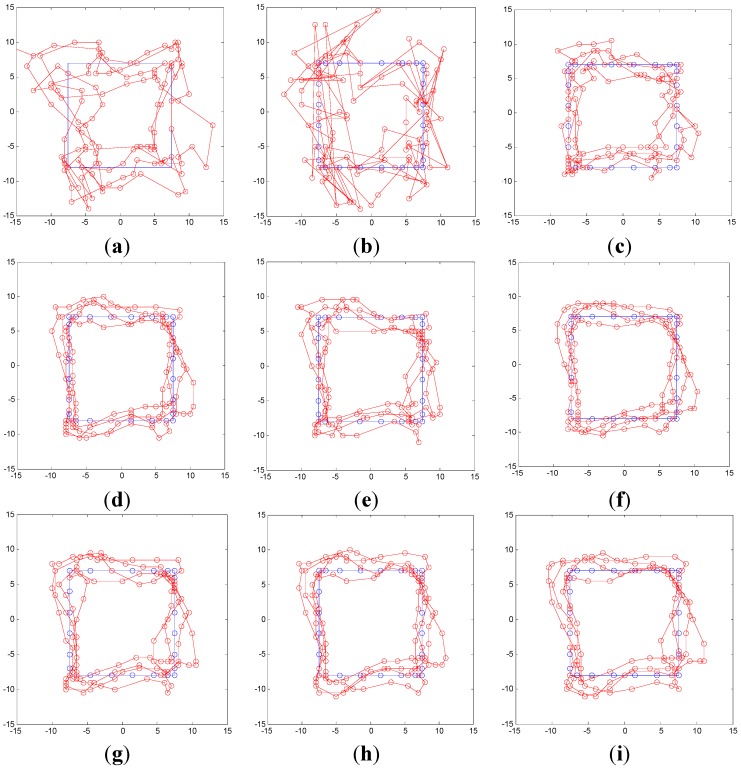
Simulation trajectories of different algorithms. (**a**) EKF; (**b**) ML; (**c**) D/TA; (**d**) I-D/TA; (**e**) PF; (**f**) M-HMM; (**g**) IMM-D/TA; (**h**) IMM + RM-HMM; (**i**) IMM + M-HMM.

The simulation results are shown in Figure 18. The lines represent the same quantities as in Section V-A. The figures compare the true trajectory (blue line) with that calculated (red line) using (a) EKF estimation; (b) ML estimation; (c) D/TA estimation; (d) I-D/TA estimation; (e) PF estimation; (f) M-HMM estimation; (g) IMM + D/TA estimation; (h) IMM + RM − HMM estimation; and (i) IMM + M-HMM estimation. It is clear that ML estimation exhibits the worst performance in localization, whereas IMM + RM-HMM has the best results, which are similar in precision to IMM + M-HMM. 

**Figure 19:**
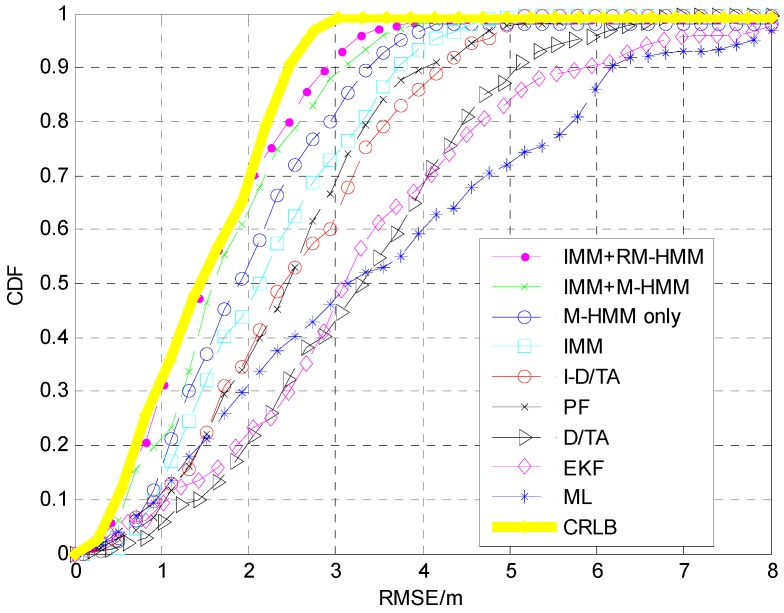
CDF analysis of various algorithms.

The CDF of each of these seven algorithms are shown in Figure 19. It is clear that the ML estimation is significantly disturbed, and PF, I-D/TA, and M-HMM show more precise results in increasing order. On this basis, IMM combined I-D/TA and M-HMM. The resulting model further improved the precision of the results, which shows the effectiveness of IMM. It is worth noting that the result obtained from M-HMM is better than that obtained using IMM + I-D/TA. RM-HMM is the best algorithm, and is a modified form of IMM + M-HMM.

**Figure 20:**
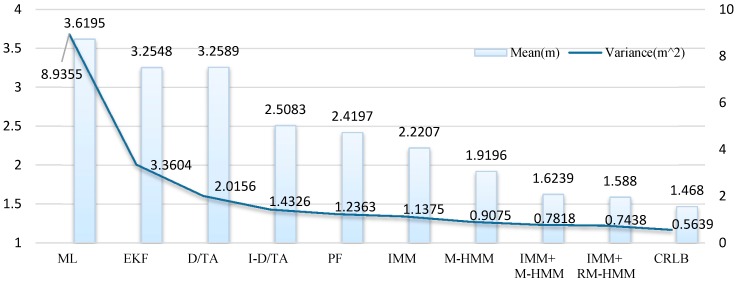
Means and variances of the nine algorithms.

Figure 20 shows that accuracy and stability improved every time the algorithm improved. For a single reduction in locating error, I-D/TA exhibits the best performance. EKF performs the best in improving locating stability. Although the improvements resulting from later algorithms are smaller, they improve the accuracy by approximately 10%. In comparison with M-HMM, the accuracy of RM-HMM is closer to that of CRLB.

**Figure 21:**
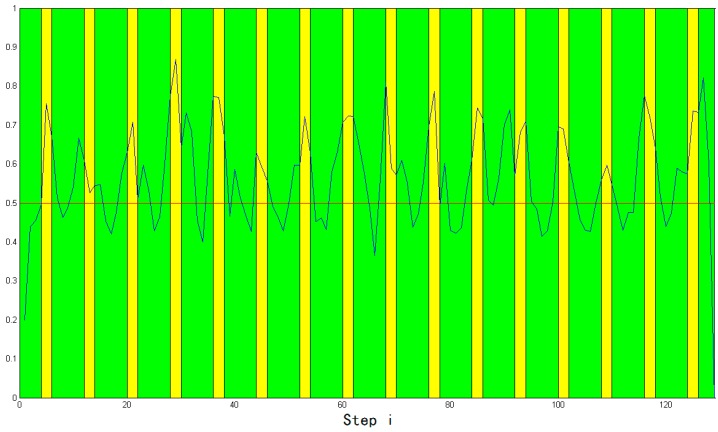
Transformation of patterns in the simulation.

The IMM state in IMM + M-HMM is shown in Figure 21. In this paper, the MT interconverts between the low-speed and high-speed situations. To show this operation more intuitively, the probability of the MT occurring in the high-speed mode is shown in Figure 21 using a blue line with an initial value of 0.2. In the figure, the green background represents the acceleration and deceleration, whereas the yellow background denotes uniform velocity. The red line is a line of reference with probability 0.5. From these data, it can be seen that the probability fluctuates with changing colors, and virtually all the peaks of the blue line match the yellow area, which means that the high-speed model was well selected. On the other hand, the bottom of the blue line matches the green area, which implies that the low-speed model was also well chosen.

**Figure 22:**
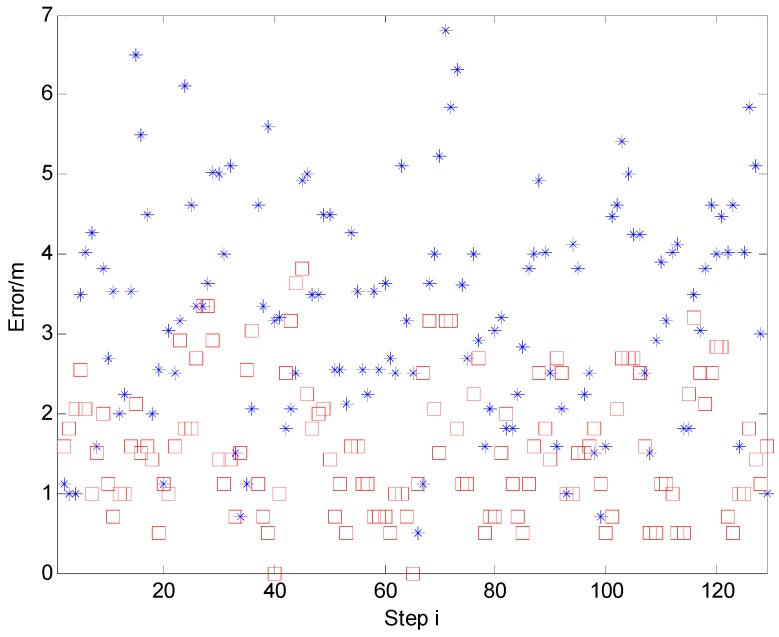
The error distribution of the improved algorithm.

Figure 22 shows the error distribution of D/TA and IMM + RM-HMM. The blue star represents the D/TA error and the red square represents the IMM + RM-HMM’s. It can be seen that the algorithm proposed above improves the estimate performance in robustness and precision generally. The analysis of complexity of the algorithms above is shown in Appendix B.

**Figure 23:**
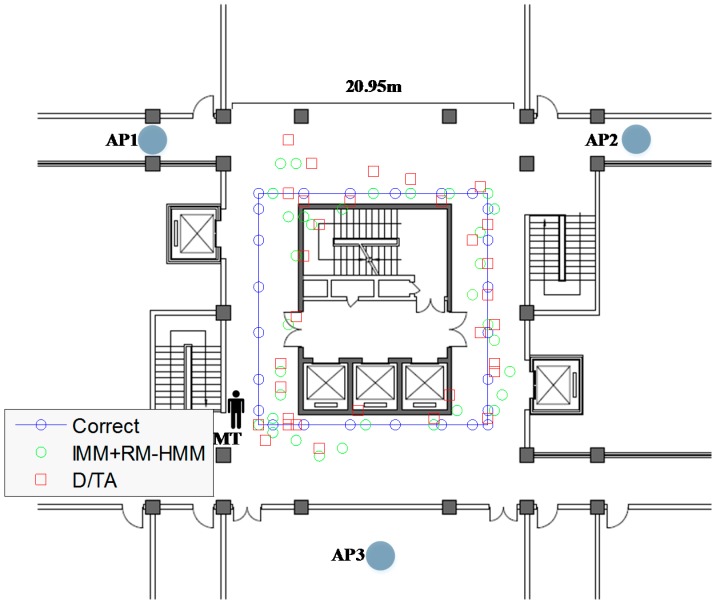
The trajectory of the MT in simulation.

A real experiment is done in the third floor of the Comprehensive Technical Building of Northeastern University to show the effectiveness of the algorithm in complex indoor environments. For comparison purposes, the trajectory of MT is as the same as before, so are the parameters. The AP1 and AP2 are put in the corridor of the building, and the AP3 is put into a room and each AP is bound on a 1.5 meters tall table tripod. Some important signs are put on the ground of the aisle based on the calculation and measurement as shown as blue circle in Figure 23. A person takes a mobile node walking along the mark points to receive the signal from the three APs. After rounding one lap, the received data are transferred into the computer server with 16 cores and 32GB RAM to estimate the trajectory of MT. 

To illustrate the effectiveness of such methods, we set up two experiments in the described scenario and we analyze the results both in simulation and practice environment. The mean value of localization error is found to be 2.73 m in D/TA and 1.69 m in IMM combined with RM-HMM for the simulation respectively. Although the layout of the APs is different from before, the algorithms still show similar precision of localization. In Figure 23, green circle represents the trajectory of IMM + RM-HMM, and the red square represents the trajectory of D/TA.

**Figure 24:**
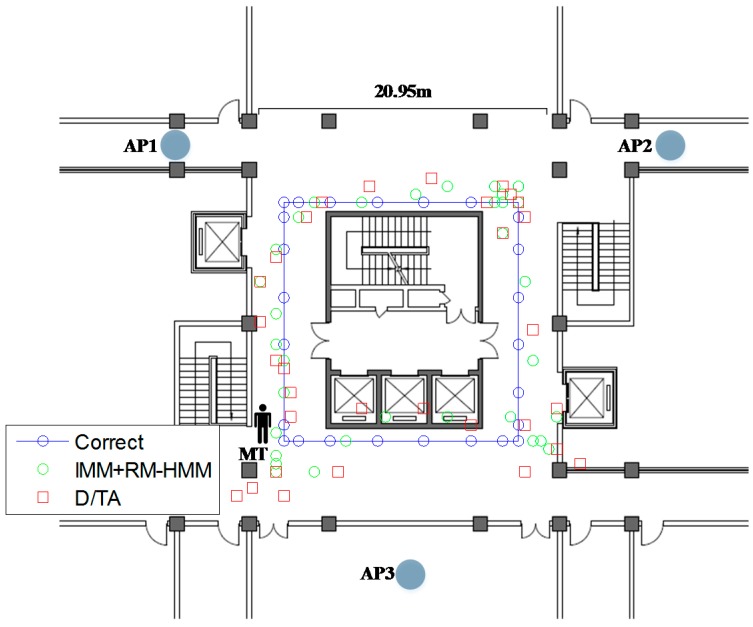
The trajectory of the MT in practice.

The practice experiments trajectory is shown in Figure 24. The legends are similar to the ones in Figure 23. The new mean values of localization errors are found to be 3.49 m in D/TA and 2.08 m in IMM combined with RM-HMM for the practice experiments. 

As can be verified from the scenario, the presence of five big metallic elevators, several power transformation boxes in the wall and metal bookcases cause severe degradation to the accuracy of the localization system in practice. It is worth mention that these phenomena are not reflected in simulation experiment. As is expected, localization with IMM + RM-HMM results in more accurate and stable locations than D/TA.

## 8. Conclusions

In this paper, we focused on WSN localization in LOS and NLOS mixed situations using an improved HMM algorithm. We first defined the concept of a UWB computation module and the HMM model. We then proposed a novel approach to locate the positions of an MT in dense multipath indoor environments. We proposed a Modified-HMM (M-HMM) method and a Replacement Modified-HMM (RM-HMM) method, and compared them with the D/TA, I-D/TA, PF, ML estimation algorithms. An IMM model was subsequently used to combine the low-velocity model with the high-velocity model in order to render the algorithm more robust and further improve precision. Simulation and real experiment results showed that our proposed methods can improve both the precision and the stability of localization. The location error was very close to the limit of CRLB.

## Figures and Tables

**Figure A1:**
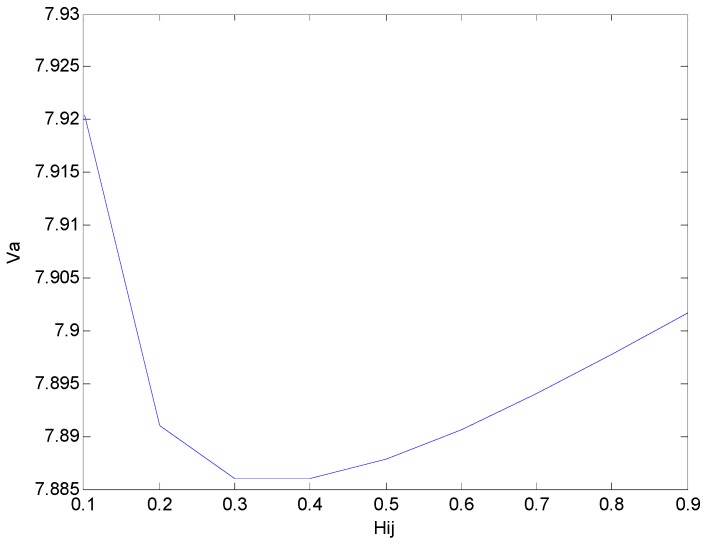
Relationship between *h_ij_* and *Va*.

**Figure A2:**
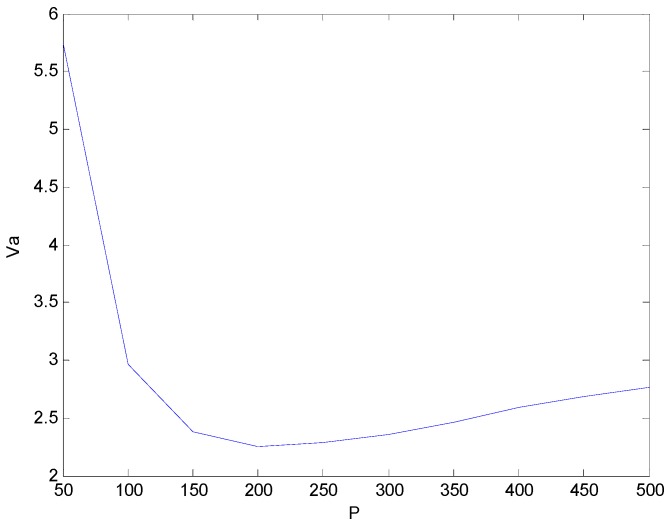
Relationship between P and *Va*.

## Appendix

### A. Analysis of Complexity

*Va* is defined as the result value of the integral of Equation (5) minus the integral of exponential probability density function σ_δ_^‒1^*exp*(‒τ/σ_δ_) with σ_δ_ *=* 7, and the data range of integral is the integer from 0 to 100. It is obvious that the smaller *Va* is, the better the curve fitting will be. Firstly, the value of *P* is supposed to be 400, then the relationship between *h_ij_* and *Va* is shown as follows.

From Figure A1, we can see that the *Va* has the minimum value when the *h_ij_* equals 0.3 or 0.4. As the right side of the curve is more flat, the *h_ij_* is selected to be 0.4. After that the relationship between P and *Va* is analysed in Figure A2.

From Figure A2, we can find that P = 200 is the best choice when *h_ij_ =* 0.4.

### B. Analysis of Complexity

From the reference [38], the notation used is as follows:

*N*: total number of unique states in HMM set;
*T*: number of observations.

The complexity of the standard forward, backward, Viterbi algorithms are normally given as *O*(*N*^2^*T*), so the complexity of D/TA is *O*(*N*^2^*T*). In consideration of the M-HMM and RM-HMM both contain with forward and backward process, so the complexity of them is *O*(*N*^4^*T*^2^). The IMM algorithm in this paper is essentially a Markov model with two states, and the complexities of both high-velocity and low-velocity models are *O*(*N*^4^*T*^2^), so the whole complexity of RM-HMM combined IMM can be approximated as *O*(2 × *N*^4^*T*^2^).

In order to reduce complexity of the mentioned algorithm, we have to reduce the value of N and *T*. *N* relates to the number of discrete points in the area *Q* and the number of APs. In this paper, the region is divided into a lot of grid points with Δ*d* = 0.5 m which can effectively reduce the value of *N*, so does the selection of three Aps. In order to reduce the value of *T*, we also disperse the signal value into integer number, and ignore the decimal part. We use MATLAB to assess all the computing time mentioned in this paper as follows in Table B1.

**Table B1:** Computing time of different algorithms.

Algorithm	ML	EKF	D/TA	I-D/TA	PF(400)
Time	77s	34s	155s	167s	172s
Algorithm	PF (1000)	IMM	M-HMM	IMM + M-HMM	IMM + RM-HMM
Time	488s	270s	290s	370s	396s

The “PF(400)” means PF’s particle number is 400. These algorithms are used to calculate the same trajectory of the MT with 30 steps, in the same computer server. It is deserve to be mentioned that computing time of every method is not only based on different algorithm structures, but also based on different efficiency of programming.

## References

[1] Xia D., Vlajic N. Near-optimal node clustering in wireless sensor networks for environment monitoring. Proceedings of the 2006 Canadian Conference on Electrical and Computer Engineering.

[2] Virone G., Wood L., Selavo Q., Cao L., Fang T., Doan Z.H., R. Stoleru S.L., Stankovic J.A. An advanced wireless sensor network for health monitoring. Proceedings of the 1st Transdisciplinary. Conference on Distributed Diagnosis and Home Healthcare (D2H2).

[3] Lee S.H., Lee S., Song H., Lee S.H. Wireless sensor network design for tactical military applications: Remote large-scale environments. Proceedings of the IEEE Military Communications Conference.

[4] Ares Zurita B., Park P.G., Fischione C., Speranzon A., Johansson K.H. On power control for wireless sensor networks: System model, middleware component and experimental evaluation. Proceedings of the 2007 European Control Conference (ECC).

[5] Gu Y., Lo A., Niemegeers I. (2009). A survey of indoor positioning systems for wireless personal networks. Commun. Surv. Tutor. IEEE.

[6] Liu H., Darabi H., Banerjee P., Liu J. (2007). Survey of wireless indoor positioning techniques and systems. IEEE Trans. Syst. Man Cybern. Part C Appl. Rev..

[7] Drawil N.M., Amar H.M., Basir O. (2013). GPS localization accuracy classification: A context-based approach. IEEE Trans. Intell. Transp. Syst..

[8] Zhong Z., He T. MSP: Multi-sequence positioning of wireless sensor nodes. Proceedings of the 5th International Conference on Embedded Networked Sensor Systems.

[9] Zhong Z., He T. Achieving range-free localization beyond connectivity. Proceedings of the 7th ACM Conference on Embedded Networked Sensor Systems.

[10] Catovic A., Sahinoglu Z. (2004). The Cramér–Rao bounds of hybrid TOA/RSS and TDOA/RSS location estimation schemes. IEEE Commun. Lett..

[11] Ribeiro A., Schizas I.D., Roumeliotis S., Giannakis G.B. (2010). Kalman filtering in wireless sensor networks. IEEE Control Syst..

[12] Chen H.Y., Deng P., Xu Y.J., Li X.W. A robust location algorithm with biased extended Kalman filtering of TDOA data for wireless sensor networks. Proceedings of the IEEE International Conference on Wireless Communications, Networking, and Mobile Computing.

[13] Cheng L., Wu H., Wu C., Zhang Y. (2013). Indoor mobile localization in wireless sensor network under unknown NLOS errors. Int. J. Distrib. Sens. Netw..

[14] Caballero F., Merino L., Maza I., Ollero A. A particle filtering method for wireless sensor network localization with an aerial robot beacon. Proceedings of the IEEE International Conference on Robotics and Automation (ICRA).

[15] Vera R., Ochoa S., Aldunate R. (2011). EDIPS: An Easy to Deploy Indoor Positioning System to support loosely coupled mobile work. Person. Ubiquit. Comput..

[16] Morelli C., Nicoli M., Rampa V., Spagnolini U. (2007). Hidden Markov models for radio localization in mixed LOS/NLOS conditions. IEEE Trans. Signal Process..

[17] Chen B.S., Yang C.Y., Liao F.K., Liao J.F. (2009). Mobile location estimator in a rough wireless environment using extended Kalman-based IMM and data fusion. IEEE Trans. Veh. Technol..

[18] Chan Y.T., Tsui W.Y., So H.C., Ching P.C. (2006). Time-of-arrival based localization under NLOS conditions. IEEE Trans. Veh. Technol..

[19] Heidari M., Alsindi N.A., Pahlavan K. (2009). UDP identification and error mitigation in TOA-based indoor localization systems using neural network architecture. IEEE Trans. Wirel. Commun..

[20] Yu K., Dutkiewicz E. (2013). NLOS identification and mitigation for mobile tracking. IEEE Trans. Aerosp. Electro. Syst..

[21] Chen P.C. A non-line-of-sight error mitigation algorithm in location estimation. Proceedings of the IEEE Wireless Communications and Networking Conference (WCNC).

[22] Marano S., Gifford W.M., Wymeersch H., Win M.Z. Nonparametric obstruction detection for UWB localization. Proceedings of the 2009 IEEE Global Telecommunications Conference.

[23] Marano S., Gifford W.M., Wymeersch H., Win M.Z. (2010). NLOS identification and mitigation for localization based on UWB experimental data. IEEE J. Sel. Areas Commun..

[24] Wang J., Gao Q.H., Yu Y., Wang H.Y. (2012). Toward robust indoor localization based on Bayesian filter using chirp-spread-spectrum ranging. IEEE Trans. Ind. Electron..

[25] Merino L., Caballero F., Ollero A. Active sensing for range-only mapping using multiple hypothesis. Proceedings of the IEEE/RSJ International Conference on Intelligent Robots and Systems (IROS).

[26] Wang Q., Balasingham I., Zhang M., Huang X. (2011). Improving RSS-based ranging in LOS-NLOS scenario using GMMs. IEEE Commun. Lett..

[27] McGuire M., Plataniotis K.N., Venetsanopoulos A.N. (2003). Location of mobile terminals using time measurements and survey points. IEEE Trans. Veh. Technol..

[28] Nicoli M., Morelli C., and Rampa V. (2008). A jump Markov particle filter for localization of mobile terminals in multipath indoor scenarios. IEEE Trans. Signal Process..

[29] Liao J.F., Chen B.S. (2006). Robust mobile location estimator with NLOS mitigation using interacting multiple model algorithm. IEEE Trans. Wirel. Commun..

[30] Yang C.Y., Chen B.S., Liao F.K. (2010). Mobile location estimation using fuzzy-based IMM and data fusion. IEEE Trans. Mob. Comput..

[31] Hammes U., Zoubir A.M. (2011). Robust MT tracking based on M-estimation and interacting multiple model algorithm. IEEE Trans. Signal Process..

[32] Qi Y., Kobayashi H., Suda H. (2006). Analysis of wireless geolocation in a non-line-of-sight environment. IEEE Trans. Wirel. Commun..

[33] Huang J., Wang P., Wan Q. (2011). CRLBs for WSNs localization in NLOS environment. EURASIP J. Wirel. Commun. Netw..

[34] Yin F., Fritsche C., Gustafsson F., Zoubir A.M. (2013). TOA-based robust wireless geolocation and Cramér-Rao lower bound analysis in harsh LOS/NLOS environments. IEEE Trans. Signal Process..

[35] Morelli C., Nicoli M., Rampa V., Spagnolini U. Particle filters for RSS-based localization in wireless sensor networks: An experimental study. Proceedings of the 2006 IEEE International Conference on Acoustics, Speech and Signal Processing.

[36] Ru J., Wu C., Zhang Y., Gong R., Liu P. (2013). Improved D/TA and information fusion based on HMM indoor localization. Appl. Mech. Mater..

[37] Spagnolini U., Rampa V. (1999). Multitarget detection/tracking for monostatic ground penetrating radar: Application to pavement profiling. IEEE Trans. Geosci. Remote Sens..

[38] Johnson MT. (2005). Capacity and complexity of HMM duration modeling techniques. IEEE Signal. Process. Lett..

